# Bilateral intermediate uveitis following treatment with paclitaxel in a patient with invasive ductal carcinoma of the breast

**DOI:** 10.1186/s40942-022-00415-y

**Published:** 2022-09-06

**Authors:** Michael Kvopka, Justine R. Smith, Bogda Koczwara, Stewart R. Lake

**Affiliations:** 1 Ophthalmology Unit, Division of Surgery, Flinders Medical Centre, Adelaide, Australia; 2 Flinders University College of Medicine and Public Health, Adelaide, Australia; 3 Department of Medical Oncology, Flinders Medical Centre and Flinders University College of Medicine and Public Health, Adelaide, Australia; 4 Eye & Vision Health, Flinders University College of Medicine and Public Health, Flinders Medical Centre Room, Flinders Drive, Bedford Park, SA 5042 Australia

**Keywords:** Paclitaxel, Taxane, Chemotherapy, Uveitis, Intermediate uveitis

## Abstract

**Background:**

To report a case of bilateral intermediate uveitis without cystoid macular edema secondary to paclitaxel therapy, and its successful management with oral corticosteroids.

**Case presentation:**

A 66-year-old female developed bilateral intermediate uveitis with reduced best corrected visual acuity to 20/40 right and 20/200 left, following 12 cycles of paclitaxel therapy for breast carcinoma. Optical coherence tomography demonstrated no cystoid macular edema in either eye, and fundus fluorescein angiography showed localized retinal vascular leakage. Resolution of uveitis and improvement of visual acuity followed treatment with oral prednisolone for two months. Fourteen months after presentation, right and left visual acuities had returned to 20/32 and 20/40, respectively, and there was no recurrence of the uveitis.

**Conclusions:**

This is the first reported case of bilateral intermediate uveitis in a patient treated with paclitaxel. Drug-induced uveitis should be considered in patients with visual symptoms in the setting of taxane chemotherapy, and oral corticosteroids are a safe and effective treatment.

## Background

Paclitaxel is a chemotherapeutic agent of the taxane class, used in the treatment of multiple cancers, including breast, lung, and ovarian carcinoma. The drug inhibits cell proliferation by binding to the β-subunit of tubulin and hyper-stabilizing the microtubules during mitosis [1], thus preventing disassembly of microtubule complexes and causing mitotic arrest [2]. Non-ocular taxane-associated adverse effects include, but are not limited to, hypersensitivity reactions, myelosuppression and immunocompromise, neurotoxicity, cardiac toxicity, and myalgia [3]. Ocular side effects are seen in 1.1% of patients receiving taxanes [4].

We report the presentation and clinical course of a 66-year-old female who presented with bilateral intermediate uveitis following paclitaxel chemotherapy for invasive ductal carcinoma of the breast. Resolution of the uveitis was achieved with oral prednisolone.

## Case presentation

A 66-year-old female presented to the ophthalmic emergency clinic with vision loss and irritation in the left eye 5 weeks after completing her final cycle of chemotherapy for early-stage breast cancer. She reported 3 weeks of watery discharge from the right eye, and 5 days of a red, itchy left eye, with left visual disturbance and floaters for 4 days. She had self-medicated with chloramphenicol 0.5% eye drops without benefit. She had a history of invasive ductal carcinoma of the breast for which a left partial mastectomy had been performed. There were no metastases reported in sentinel nodes, but adjuvant chemotherapy and radiotherapy were recommended. Five weeks prior to presentation she had completed 4 cycles of doxorubicin and cyclophosphamide, followed by 12 cycles of paclitaxel. She experienced peripheral neuropathy of the fingers and toes 7 weeks after beginning chemotherapy; this had improved since cessation of paclitaxel. She had no other past medical or ocular history, and she was not taking any regular medications.

**Fig. 1:**
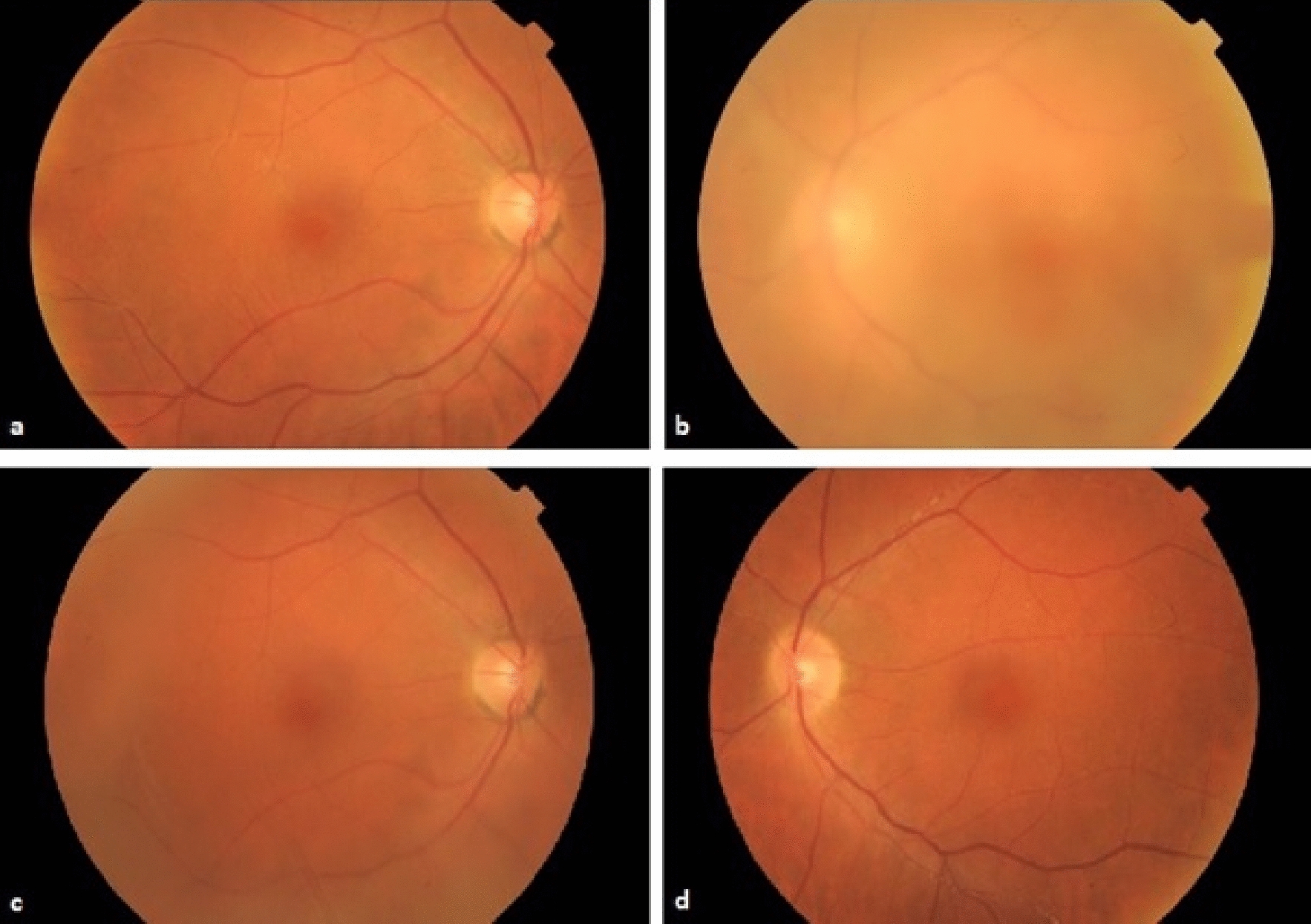
**a**–**d** Color fundus photographs. **a** Right eye fundus taken at time of presentation, **b** left eye fundus at time of presentation demonstrating vitreous haze, **c** right eye fundus 1 month after presentation with interval development of vitreous opacity, **d** left eye fundus at 1 month after presentation, following PPV

**Fig. 2:**
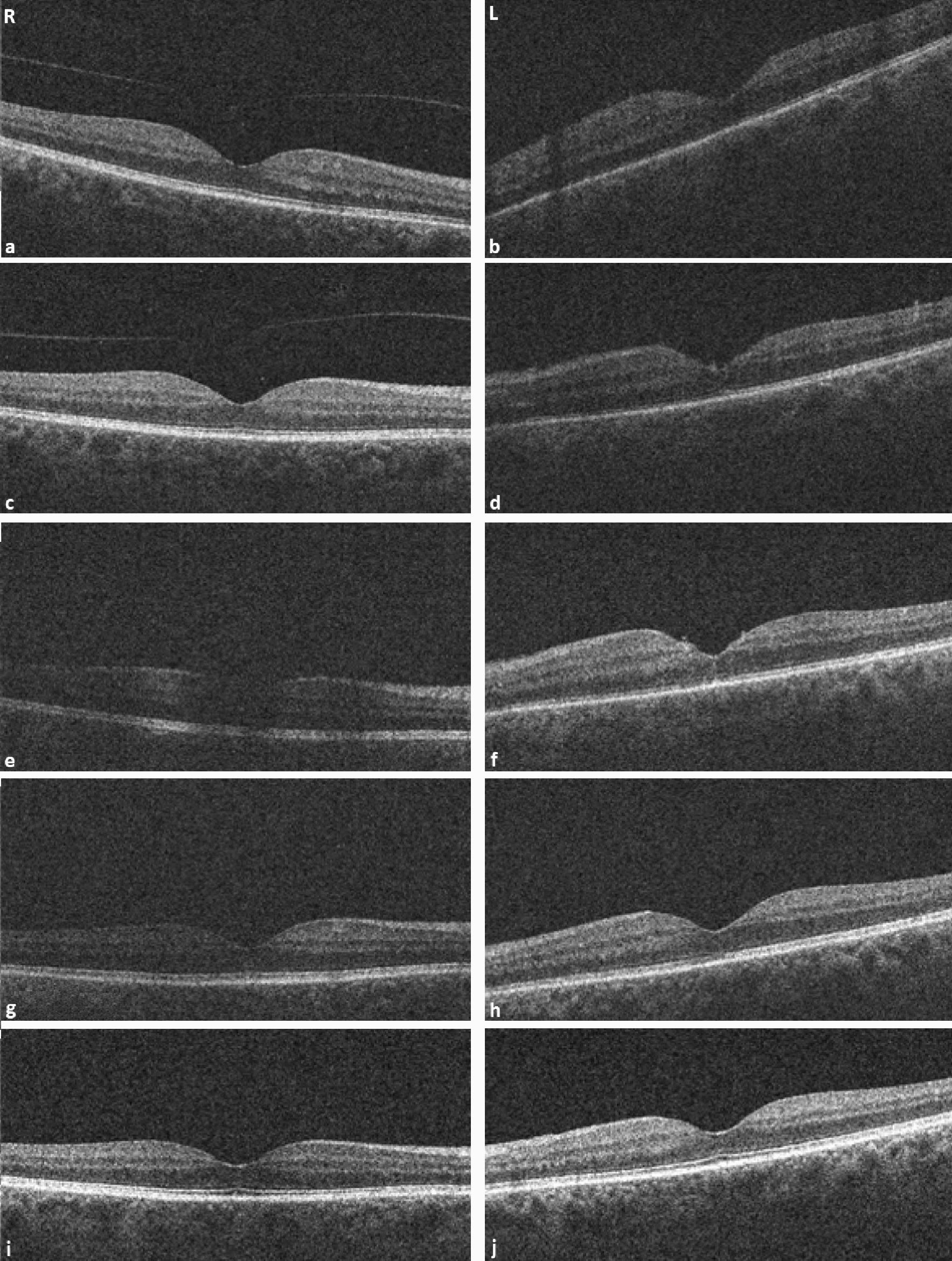
**a–j** Optical coherence tomography (OCT) of the maculae. **a**, **b** At presentation. **c**, **d** One day after presentation. **e**, **f** At 2 weeks following presentation. Oral prednisolone was commenced. **g**, **h** At 1 month after presentation and 2 weeks after starting oral prednisolone. **i**, **j** At 14-month follow-up. No CME was seen at presentation or at any point during follow-up

**Fig. 3:**
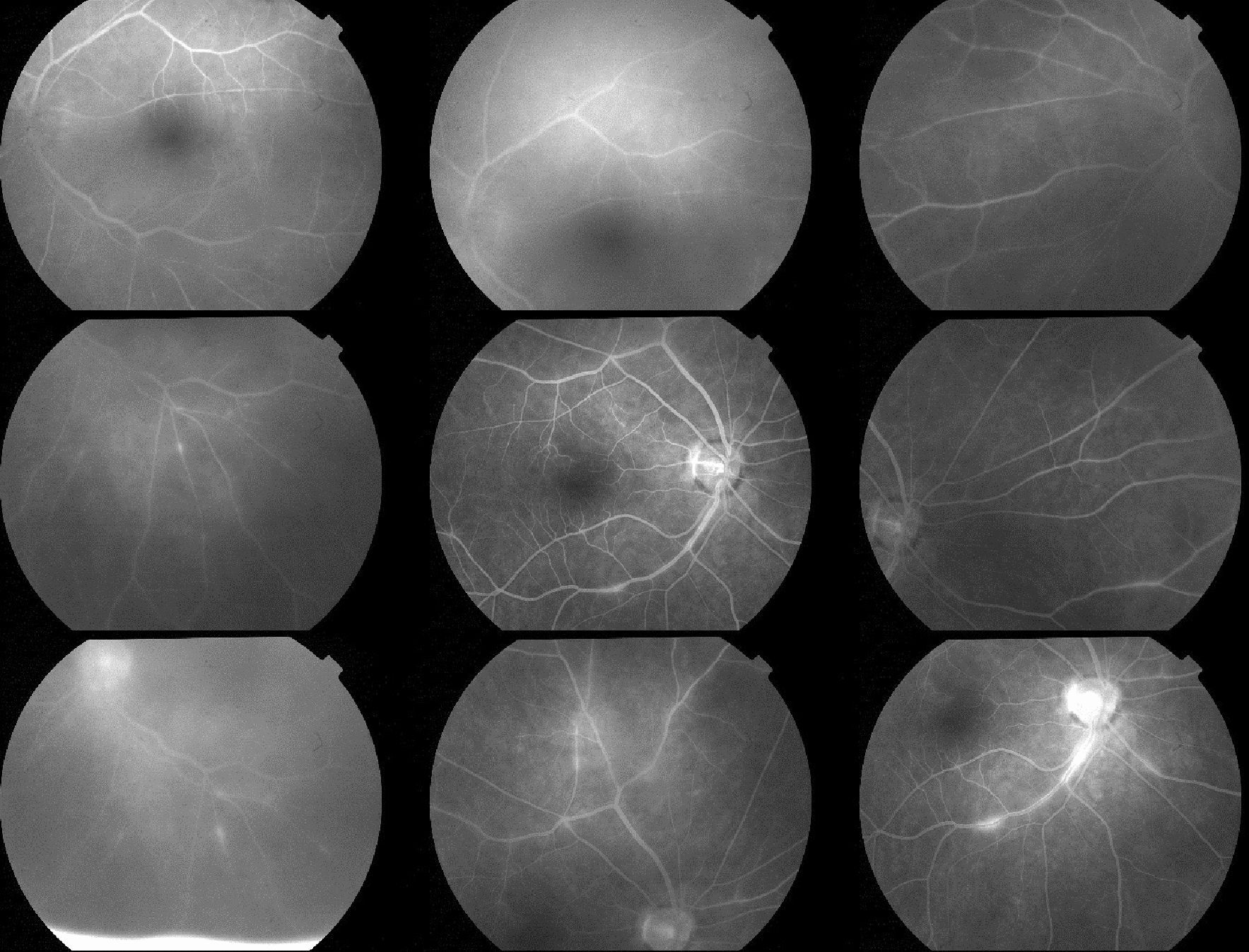
Late phase fundus fluorescein angiographic survey taken at presentation. Bilateral multi-focal retinal venous wall staining with leakage is seen in both eyes. Cystoid macular edema is absent from all images

On examination, best corrected visual acuities (BCVA) were 20/25 on the right and 20/80 on the left. Superficial punctate epithelial erosions were present in both eyes, and the left eye had fine keratic precipitates, grade 1+ anterior chamber cells, grade 2+ anterior chamber flare, posterior synechiae, vitreous cells, grade 2+ vitreous haze (Fig. 1) and retinal vascular sheathing. Optical coherence tomography (OCT) confirmed no cystoid macular edema (CME) (Fig. 2). Fundus fluorescein angiography demonstrated retinal vessel wall staining with predominantly venous leakage in both eyes (Fig. 3).

One week after presentation, right BCVA was 20/25 and left BCVA was 20/200, and there had been no change in the intraocular inflammatory signs. Serum c-reactive protein and angiotensin-converting enzyme were within normal limits, and her chest x-ray was normal. Syphilis IgG, antinuclear antibodies, and antineutrophil autoantibodies were negative. The Quantiferon Gold interferon-gamma release assay, and serological tests for *Toxoplasma gondii* and *Treponema pallidum* were negative. Diagnostic left anterior chamber paracentesis and pars plana vitrectomy were performed. Microscopy and culture of both aqueous and vitreous were negative for bacteria, including acid-fast bacilli, and fungi, and polymerase chain reaction of the samples did not detect cytomegalovirus, enterovirus, herpes simplex virus 1 and 2, varicella-zoster virus, parechovirus, *Neisseria meningitidis*, and *Streptococcus pneumoniae*. Cytology and immunophenotyping did not support a diagnosis of vitreoretinal lymphoma.

The patient was commenced on prednisolone acetate 1% drops (Pred Forte) 4-times daily, and cyclopentolate 1% once daily to both eyes; valaciclovir was continued whilst awaiting test results. Five days following the vitrectomy, left BCVA had improved to 20/50, but right BCVA was reduced to 20/40. At this time the right eye had vitreous cells and grade 2+ vitreous haze (Fig. 1), with scattered retinal vascular sheathing, while the left eye had grade 0 vitreous haze, with persistent retinal vascular sheathing. A diagnosis of bilateral intermediate uveitis and retinal vasculitis associated with paclitaxel was made. The patient was switched to oral prednisolone 60 mg (1 mg/kg) once daily. By this stage she had developed toenail and fingernail onycholysis and hyperkeratosis. Fungal scrapings of her nails were negative.

One week later, BCVAs were 20/40 and 20/60 on the right and left respectively. Right eye demonstrated grade 0.5+ anterior chamber cells, grade 0 anterior chamber flare, and grade 3+ vitreous haze; left eye anterior and posterior segments were normal, with resolution of the retinal vessel sheathing. Within one month of starting oral prednisolone, the patient reported a substantial subjective improvement in her ophthalmic symptoms, and the vitreous inflammation had resolved. Oral prednisolone was weaned from 60 mg and ceased over 4 months. Two months after stopping prednisolone there was no uveitis in either eye. The patient continues to be reviewed annually, and at most recent follow-up 15 months after ceasing treatment, right BCVA was 20/32 and left BCVA was 20/40, with normal anterior and posterior segments in both eyes.

## Discussion and conclusions

This case is the first reported bilateral intermediate uveitis, which notably developed no CME, in a patient treated with paclitaxel. Our patient had a negative infectious and inflammatory retinal vasculitis workup. The literature searched for paclitaxel-induced uveitis included the MEDLINE database, FDA adverse events databases, and Google. Drug-induced uveitis is known, but uncommon, cause of uveitis [5]. A recent study of non-infectious uveitis in the US indicated a prevalence of 121 cases in 100,000, with a specific prevalence of non-infectious panuveitis of 12 cases per 100,000 persons [6]. Drug-induced uveitis accounts for less than 0.5% of referrals to tertiary uveitis clinics [5]. Drugs which have been associated with uveitis can broadly be categorised by their respective methods of administration: systemic, topical, and intraocular. Systemic treatments judged to be definitive causative agents of uveitis included rifabutin, bisphosphonates, and sulphonamides; those graded as probable causative agents included fluoroquinolones, diethylcarbamazine, and tumor necrosis factor-α inhibitors [7]. Newer research has also implicated immune checkpoint inhibitors, and BRAF/MEK inhibitors as definitive, systemically-administered, causes of uveitis [8]. Taxanes have not been previously reported as inducers of uveitis.

Paclitaxel-based chemotherapy is known to cause ocular side effects including meibomian gland dysfunction [4], canalicular obstruction [4], diplopia [4], keratitis [9], CME [10], scintillating scotoma [11], and possibly glaucoma [12, 13]. Das et al*.* recently reported bilateral severe ischaemic retinopathy and optic neuropathy in a 72-year-old patient treated with combination cyclophosphamide and paclitaxel therapy for breast carcinoma [14]. Their patient developed significant bilateral CME, which our patient did not have [14]. CME appears to be the most common retinal complication of both paclitaxel and docetaxel, despite occurring at a very low rate amongst treated patients [15]. It is often bilateral, and can be angiographically silent [10, 16–18]. The CME occurs several months after starting paclitaxel treatment, consistent with the timeline of uveitis onset in our patient. Our patient also developed toenail and fingernail hyperkeratosis, onycholysis, and a single complete toenail loss at the time of intermediate uveitis, findings which are consistent with a paclitaxel-related toxicity [19–21]. She also experienced neurotoxicity in the form of peripheral neuropathy, a known side effect of paclitaxel [22]. A test–retest was not performed for our patient as the delayed onset of side effects presented a diagnostic challenge, and further treatment with paclitaxel was not indicated given the patient had already completed the course of chemotherapy. Although the retinal appearance was not typical of acute retinal necrosis, our patient was initially treated with valaciclovir as a precaution, given immunocompromise is a risk factor for an infectious uveitis [23].

Paclitaxel toxicity in our patient was manifested in the form of nail changes, peripheral neuropathy, and bilateral intermediate uveitis. Our patient demonstrated a temporal association between intermediate uveitis and paclitaxel therapy. Her rapid remission of bilateral intermediate uveitis with a limited course of systemic corticosteroids, absence of CME (atypical for non-infectious panuveitis), and negative testing for other causes of uveitis all are suggestive of drug-induced uveitis secondary to paclitaxel therapy.

Bilateral intermediate uveitis may be a rare adverse effect of chemotherapy with paclitaxel. Treating oncologists should screen patients for ophthalmic symptoms. Reduced vision during or soon after treatment with paclitaxel warrants an urgent ophthalmic review.

## Data Availability

Not applicable.
